# Chemical modulation of autophagy as an adjunct to chemotherapy in childhood and adolescent brain tumors

**DOI:** 10.18632/oncotarget.26186

**Published:** 2018-10-16

**Authors:** Juliette Servante, Jasper Estranero, Lisethe Meijer, Rob Layfield, Richard Grundy

**Affiliations:** ^1^ School of Life Sciences, University of Nottingham, Medical School, Nottingham, NG7 2UH, UK; ^2^ Children’s Brain Tumor Research Centre, Medical School, Queen’s Medical Centre, Nottingham, NG7 2UH, UK

**Keywords:** autophagosome, autophagy, childhood brain tumors, chloroquine, mTOR

## Abstract

Brain tumors are the leading cause of cancer-related death in children and are the most challenging childhood cancer in relation to diagnosis, treatment, and outcome. One potential novel strategy to improve outcomes in cancer involves the manipulation of autophagy, a fundamental process in all cells. In cancer, autophagy can be thought of as having a “Janus”-like duality. On one face, especially in the early phases of cancer formation, autophagy can act as a cellular housekeeper to eliminate damaged organelles and recycle macromolecules, thus acting as tumor suppressor. On the other face, at later stages of tumor progression, autophagy can function as a pro-survival pathway in response to metabolic stresses such as nutrient depravation, hypoxia and indeed to chemotherapy itself, and can support cell growth by supplying much needed energy. In the context of chemotherapy, autophagy may, in some cases, mediate resistance to treatment. We present an overview of the relevance of autophagy in central nervous system tumors including how its chemical modulation can serve as a useful adjunct to chemotherapy, and use this knowledge to consider how targeting of autophagy may be relevant in pediatric brain tumors.

## INTRODUCTION

Central nervous system (CNS) brain tumors are both the leading cause of cancer-related death in childhood/adolescence and the most common form of solid tumor in this age group [1]. In the US, CNS tumors have an incidence of 5.54 per 100,000 children (0-14 years of age) (2010-2014) and, despite medical advances in both earlier detection and more effective treatment, 10-year survival remains less than 75% [1]. However, this masks the outcomes in some tumor types such as diffuse intrinsic pontine gliomas (DIPG) that are invariably fatal. The mainstay of treatment of pediatric brain tumors is surgery with optional adjuvant radiotherapy or chemotherapy determined through patient age, tumor type, degree of surgical resection, location and grade [2]. Approximately 66% of survivors are left with significant disabilities that shorten survival and affect re-integration into society [3]. Further advances in the treatment of children’s brain tumors are therefore clearly needed to improve patient morbidity and mortality.

Autophagy, Greek for ‘self-eating’, is a pathway of potential interest as a target for future anti-cancer agents. It is a catabolic process that promotes cellular homeostasis through the recycling of damaged proteins and organelles. The pathway of autophagy begins with the formation of a double membrane enclosed layer around proteins/organelles for recycling. The newly formed structure (now known as an autophagosome) fuses with an acidified lysosome, promoting breakdown of its contents.

Cells require an ongoing production of proteins for their survival and this consumes much of the cellular energy supply. In times of nutrient deprivation cells need to maintain their energy levels and to achieve this, the cell employs autophagy to ‘eat’ redundant proteins and components in order to generate energy. In this manner the cell provides its own survival mechanism. Autophagy may also affect cell survival as aberrant proteins, if left, could build up within the cell and have the potential to affect signalling and transport mechanisms [4]. The process also helps to remove reactive oxygen species (ROS) that could otherwise cause gene mutation and possible loss of control of cell turnover.

The therapeutic targeting of autophagy is already under investigation for the treatment of neurodegenerative diseases and specific cancers including hepatocellular carcinoma [5], melanoma [6, 7] and breast cancers [8]. Autophagy modulation has also been considered as a treatment strategy for brain tumors both in adults and children [9–14] and although its exact role in pediatric CNS neoplasms is not yet known [9], build-up of autophagosomes is enhanced in tumors such as gangliogliomas [15]; in treatment with chemotherapy agent temozolomide (TMZ) [16]; and in association with cell death [17].

In this paper, we first explore the variable roles of autophagy in tumor evolution. The mechanism of autophagy will then be discribed with particular reference to different stages that can be regulated both physiologically and through pharmacological interventions. Current evidence for the potential of a combinatorial treatment strategy (chemotherapy with autophagy modulation) comes mostly from studies of adult tumors; this will be explored with consideration of the distinctions between adult and childhood brain tumors. Finally, we will examine the currently limited available evidence for the potential of the combinatorial strategy in targeting pediatric tumors and speculate on new avenues to explore in the future.

## THE PARADOXICAL ROLE OF AUTOPHAGY IN CELL SURVIVAL AND TUMOR EVOLUTION

Of the three different subtypes of autophagy, macroautophagy (hereafter referred to as autophagy) is the most widely investigated and is the focus of this paper. Autophagy involves the initiation and formation of double membrane vesicles, known as autophagosomes, around cellular components for degradation. Methods of autophagy detection within the cell include monitoring the formation of autophagosomes and turnover of the proteins that recruit cargo (proteins/organelles for recycling) into the autophagosome (known as autophagy receptors). However, as autophagy can be induced as part of a survival attempt in the dying cell, build-up of autophagosomes alone is not proof of a mechanism of cell death [18] as demise may occur alongside pro-survival mechanisms. Indeed, autophagy has been closely linked to apoptosis and many of their complex components are interlinking, with evidence of both antagonism and cooperation between these pathways [19, 20].

The role of autophagy in tumor evolution depends upon the stage of growth. In the initial phase of tumorigenesis, cellular autophagy could help remove ROS from the cell to prevent subsequent DNA damage which, if left, may trigger uncontrolled cell division [9]. The pathway may aid subsequent cell survival in two ways. Removing damaged proteins and organelles during tumor growth avoids their accumulation; an event which could have triggered cell death. In addition, autophagy can help maintain cellular nutrition during periods of starvation such as tumor growth preceding neovascularization [21]. Indeed, the physical location of a cell within a tumor has been shown to affect rates of autophagy in glioma cells [22].

Where cells die due to a failure of nutrient acquisition, ROS are released from necrosing tissue. This release has the knock on effect of increasing autophagic activity in neighboring cells which enhances substrate availability [23]. This mechanism is of interest as increased levels of autophagosome formation in the tumor cell niche may make cells more vulnerable to death when subsequent steps of the pathway are inhibited either via leakage of enzymes from lysosomes, known as lysosomal cell death [18], or via possible physical disruption to cell activities by numerous autophagosomes. Additional factors contributing to a possible increased rate of autophagy in tumor cells include situations of cellular distress such as starvation, the release of ROS (as mentioned above), or endoplasmic reticulum (ER) stress [23] which can arise during chemotherapy [9] or through radiotherapy (see below).

## METHODS AND EFFECTS OF AUTOPHAGY MODULATION IN TUMOR CELLS

**Figure 1:**
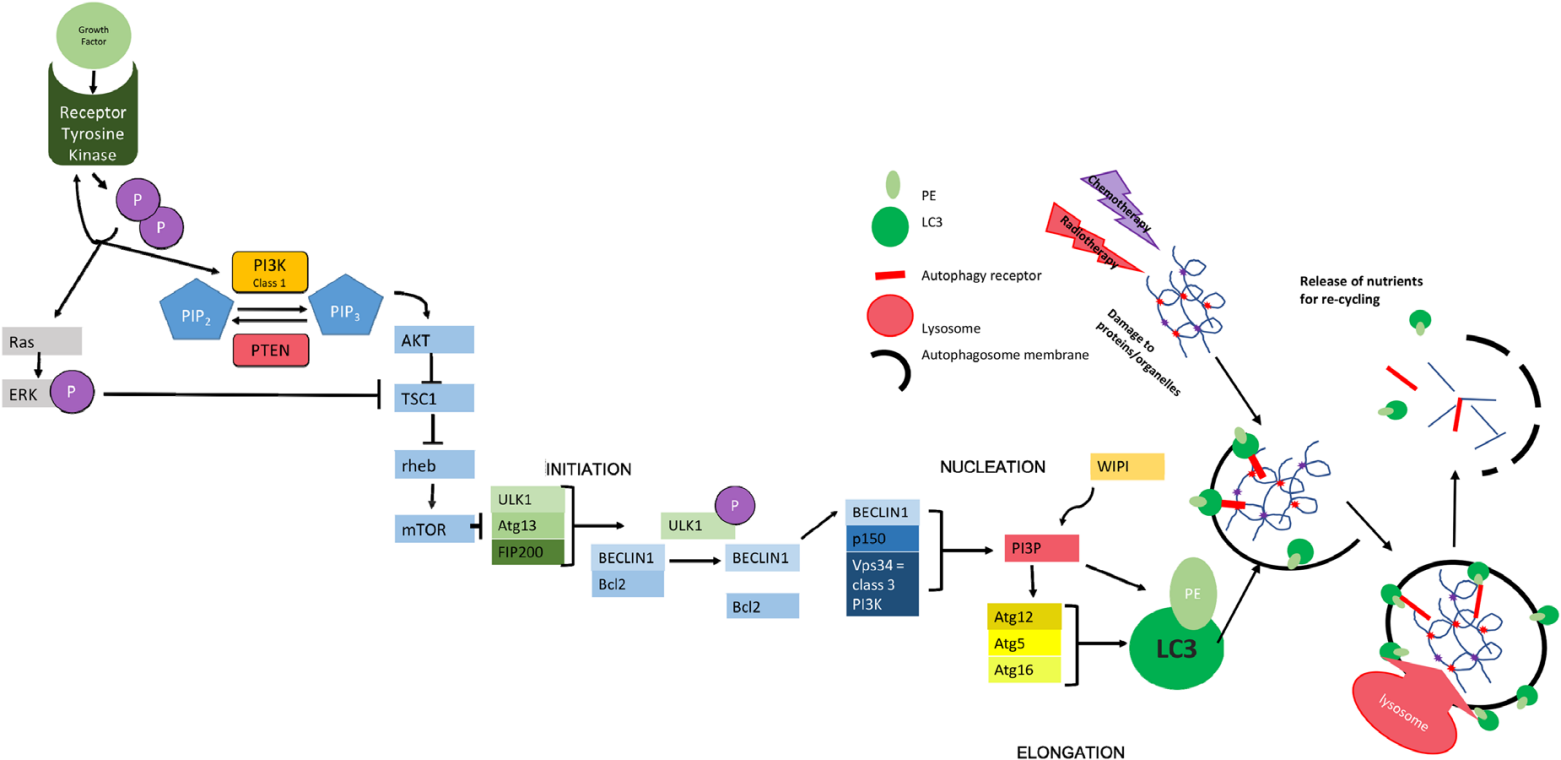
Molecular Mechanisms of Autophagy The initiation of autophagy is controlled through a series of complexes involving a group of evolutionally conserved proteins known as “autophagy related proteins” (ATGs) which eventually lead to the production of scaffold protein LC3-II; essential for autophagosome function. The initial complex involved incorporates ULK1/2 (uncoordinated 51-like kinase 1/2), ATG13 (a regulator of ULK1 auto-induction) and FIP200 (also a regulator) [38] and its assembly results in auto-phosphorylation of ATG13/ULK1. A subsequent conformational change in this preliminary complex allows the formation of further complexes [39]. The second complex formed at the site of autophagosome construction involves Beclin 1, which has been identified as a tumor suppressor [40]. Beclin 1 interacts with the anti-apoptotic regulator Bcl2 [41, 42]. This coupling is broken under situations of starvation which allows Beclin 1 to associate with VPS34 (a class 3 PI3K) and p150 (also known as VPS15) to produce PI3P. PI3P interacts with one of several WIPI proteins (WD40 repeat protein interacting with phospho-inositides) and the WIPI protein subtype determines the rate of autophagy [39, 43, 44]. In the next step of autophagy, stimulation of ATG12 allows the formation of a complex that helps in the conversion of LC3-I to LC3-II *via* the addition of phosphatidylethanolamine (PE) and in the positioning of this modified protein on the developing autophagosome [45] where it acts as a scaffold protein [46]. The specificity of autophagy comes from the involvement ofautophagy receptors, such as SQSTM1/p62, that can simultaneously bind to the autophagosomal membrane (*via* LC3) and to ubiquitin modifications used to mark autophagic targets [47]. The growing autophagosome encircles both the receptor and its target for recycling as well as other cellular waste, forming a double-membrane vesicle that is able to fuse to a lysosome either directly or *via* fusion with an endosome derivative (multi-vesicular body, MVB [15, 48]). Fusion with the lysosome allows the release of digestive enzymes into the autophagosome with consequent catabolism of proteins and organelles resulting in the release of amino acids for recycling [49]. The chief inhibitor of autophagy, mTOR works to inhibit the initial ULK1-ATG13-FIP200 complex. mTOR is a protein kinase - active when energy supply is sufficient [19, 43] - that hyperphosphorylates ATG13 and prevents auto-phosphorylation of ULK1, thereby inhibiting further steps [50, 51]. mTOR works downstream of growth factors and is also controlled by feedback of both cellular energy levels and protein availability. This includes the monitoring of amino acid levels in lysosomes using v-ATPase (vacuolar-type H+ ATPase) in the lysosomal membrane. Where levels of amino acids are sufficient, the binding of growth factors to tyrosine kinase receptors leads to receptor autophosphorylation and consequent activation of both PI3K and Ras. *Class 1* PI3K aids the phosphorylation of PIP_2_-PIP_3._, thus triggering AKT to inhibit the formation of a complex between TSC1 and TSC2. The phosphorylation of ERK by Ras also inhibits this interaction. The TSC1-2 complex normally acts to inactivate the GTPase rheb. When active, rheb upregulates mTOR activity. Therefore, action of AKT indirectly up-regulates mTOR, meaning that autophagy is inactive [52]. This is reversed during periods of starvation where nutrients are less abundant, meaning that mTOR becomes inactive and autophagy occurs at an enhanced rate. PTEN (phosphatase/tensin homolog on chromosome 10) is a phosphatase acting on lipids to cause the conversion of PIP_3_ back to PIP_2_, thus increasing levels of cellular autophagy *via* reduced mTOR activity [53].

Autophagy modulation following anti-cancer treatments can affect cell survival to both extremes. On the one hand autophagy activation acts as a protective mechanism mediating the acquired resistance phenotype of some cancer cells during chemotherapy. An example of this comes from the work that has been done using chemical inhibition of the initiation phase of autophagy (see figure 1) in neuroblastoma to sensitize cells to chemotherapy [24, 25] and the concept has also been proven in other tumor types [26–28]. In this context, inhibition of autophagy can potentially resensitise previously resistant cancer cells or augment the cytotoxicity of various chemotherapy treatments. Alternatively, autophagy induction could itself be detrimental to the cell, with cell death resulting secondary to leakage of enzymes from the lysosomes [18, 29] or due to modulation of intracellular signaling from the build up of autophagosomes [4].

Pharmacological induction of autophagy often inhibits mTOR, making autophagy constitutively more active (see Figure 1). The later stages of autophagy (i.e. the degradation of autophagosomal cargo) can also be pharmacologically controlled using chloroquine and its derivative hydroxycholoquine. (Hydroxy)Chloroquine is a drug that inhibits the fusion of autophagosomes to lysosomes [30] by increasing the lysosomal pH thus inactivating the digestive enzymes contained within it.

A simple combinatorial strategy of autophagy modulation alongside cytotoxic drug therapy was investigated by Levy and Thorburn in DAOY+ ONS76 medulloblastoma cells as well as BT-16+ BT-12 CNS atypical teratoid/rhabdoid tumor cells treated with chemotherapy agents CCNU and cisplatin. Results varied between cell types but on the whole, cell survival in the presence of cytotoxic drugs was unaffected by either autophagy activation alone using the mTOR inhibitor rapamycin or inhibition of autophagosome degradation alone using chloroquine [9]. These results suggest that autophagy modulation is not as straight-forward as initially hoped and cell response to modulation is likely to be context dependent. New therapies are likely to be specific to tumor type [31] and cell environment [22]. Notably a number of important cancer-related signalling pathways have been implicated in the regulation of autophagy. The phosphatidylinositol 3-kinase/mammalian target of rapamycin (PI3K/mTOR) and the AMP-activated protein kinase (AMPK) pathways have emerged as central conduits in the regulation of autophagy. Mutations in these pathways are associated with malignancies such as breast cancer [32, 33], ovarian cancers [34] and leukemia [35, 36] and modulation of these pathways is being considered as a target for new cancer therapies irrespective of their effects on autophagy. Caution should therefore be taken when selecting potential strategies for targeting autophagy as each target may modulate multiple pathways and have numerous conflicting roles within the cell [37]. Further work on crosstalk between these pathways may help to improve the specificity and therefore success of future compounds selected as candidate cancer treatments. Nonetheless, the combination of autophagy inhibitors with cytotoxic drugs is attracting attention, and gaps in our knowledge of the ability of autophagy manipulation to overcome resistance to anti-cancer therapies are apparent.

## EVIDENCE OF AUTOPHAGY MODULATION IN ADULT CASES

### Caveats to transferring knowledge of adult to childhood tumors

Classification of childhood brain tumors is based on the histological subtype including details of the molecular characteristics, degree of differentiation – from low (grade 1) to high grade (grade 4) according to WHO guidelines – and the original cell type. Brain tumors in childhood differ from those in adults in terms of the type of tumor, natural progression and site of occurrence [54]. Pediatric brain tumors are best considered to arise due to aberrations in normal development and are derived from embryonic structures, as is the case with the medulloblastoma. However, glioblastoma cells in adults and older children show similarities in terms of morphology and gene expression [55], suggesting that similar treatment options may be apposite. Currently, drugs like TMZ are equally ineffective as a cure for both adult and childhood/adolescent high grade glioma [56]. Overall evidence from approaches in adults is likely to be useful in directing treatments to trial in children, but on a cautionary note results may not be directly transferrable between the two distinct groups of tumor.

### Conflicting effects of 3-MA demonstrating that the outcomes of autophagy manipulation may be specific to tumor type and envionment

3-MA impedes autophagy in the early stages through the inhibition of class 3 PI3K [57] and has a variable role in glioma survival. Discrepancy in the effect of 3-MA on cell survival may come from opposing effects of simutaneous class 1 PI3K inhibition (and subsequent autophagy activation) alongside activation of class 3 PI3K due to lack of complete specificity for class of PI3K. In addition, 3-MA has been shown to act on many different pathways of metabolism within the cell. Its influence on autophagy is inconstant, with effects depending on the concentration used and the availability of nutrients.

Sun et al investigated the efficacy of berberine, an anti-bacterial agent used in China, in reducing the viability of glioma cell line LN18. Cell death was increased in treatment with berberine even where caspases were inhibited by Z-VAD, ruling out apoptosis as the mechanism. Cell death was thought to be due to a reduction in mitochondrial function and ATP availability and this was shown to occur alongside autophagy activation (demonstrated by an increase in LC3-II and decrease of autophagy receptor p62 presumed to be due to enhanced degradation). The addition of 3-MA was found to further reduce survival of these cells treated with berberine [58], and may be linked to additive function in causing cell starvation.

This is in contrast to findings that antineoplastic drug AG 1301 (1 micromole) reduced survival of C6 glioma cells to 56.67% of that of the control sample, where 3-MA enhanced cell survival. Autophagy was shown to be activated in AG1301 treatment as there was an increase in LC3-II:LC3-I with reduction in p62 [59]. Discrepancies in the effect of 3-MA on cell survival help to demonstrate that autophagy modulators as treatments may be more complicated than just their effects on autophagy and that effects are likely to be specific to tumor type, environment and concentration used.

### Autophagy inhibitor chloroquine is of potential use in the treatment of glioblastoma

Autophagy modulation as a treatment strategy has been investigated in adult glioblastoma cells using chloroquine. As noted previously chloroquine and its derivative hydroxylchloroquine inhibit lysosomal fusion to the autophagosome, an effect thought to be due to their neutralizing action as weak bases.

Glioblastoma is the most common adult malignant brain tumor and has a median survival of 14.6 months despite therapy [10]. Treatment of glioblastoma in adulthood most frequently involves alkylating agent temozolomide (TMZ) with optional radiotherapy. It was demonstrated in U87MG (glioblastoma cell line) that chloroquine treatment in addition to TMZ led to an increase in LC3-II (a marker of autophagosome formation) whilst knockout of Beclin 1 (a protein necessary in the initiation of autophagy) reduced the cytotoxic effect of chloroquine [60]. This evidence suggests an increase in autophagosome build up through additional chloroquine treatment. In a cohort of 30 glioblastoma patients aged less than 60 years, it was found that treatment with chloroquine after tumor resection as an adjunct to chemotherapy and radiotherapy increased the mean survival time to 24 months as opposed to 11 months in those who did not receive chloroquine. Following these results, chloroquine was identified as an attractive subject for study in larger cohorts [61]. A more recent study, carried out in 2015, found the addition of chloroquine to further reduce the size of C6 glioma tumors than the implementation of TMZ treatment alone [62] and is backed up by secondary findings of Min et al. whilst developing a luciferase reporting system of autophagy activity [63].

The mean tolerated dose of hydroxychloroquine in a phase 2 clinical study was found to be 600mg/day; a dose which yielded no changes in survival or tumor growth inhibition. 800mg/day resulted in neutropenia and thrombocytopenia in all three subjects, likely due to myelosuppression resulting from treatment with both TMZ and hydroxychloroquine [64]. The use of chloroquine as an adjunct to promote autophagosome build-up could theoretically be further enhanced through concurrent upregulation of autophagy using a compound such as rapamycin to enhance the initiation phase. The use of such a combination approach could reduce the therapeutic dose of (hydroxy)chloroquine. However, rapamycin can also cause neutropenia and pancytopenia [65] as can radiotherapy [66]. These are effects of most methods of chemotherapy and should be kept in mind when developing new treatment strategies.

Huang et al investigated the potential of bevacizumab, a monoclonal antibody targeting VEGF-A receptors as a treatment to induce apoptosis of U87MG glioblastoma cells. The addition of 10 micromolar chloroquine enhanced the percentage of apoptotic cells from 25.45% to 54.22%. The method of cell death from this combined approach was suggested to be due to cell starvation coming from both a lack of angiogenesis and recycling of intracellular components [67]. These findings have been reproduced by Müller-Greven et al in CD133+ve gioblastoma cells; treatment with bevacizumab was associated with enhanced autophagy levels and cell death was enhanced by adding in bafilomycinA1 (a compound that blocks lysosomal acidification). In this investigation, autophagy was demonstrated through co-localisation of LC3 puncta with a marker of lysosomes (LAMP2) [68].

### Autophagy inhibitor chloroquine is of potential use in the treatment of glioblastoma and medulloblastoma alongside fenofibrate

Fenofibrate, a PPAR alpha agonist, has been shown to force cells from a human glioblastoma cell line into B-oxidation of fatty acids. This process led to ATP depletion and AMP dependent activation of autophagy that was visualized via enhanced abundance of LC3-II. *In vitro*, the addition of 50 micromolar fenofibrate to an LN-229 glioblastoma cell culture led to 96% growth inhibition. Although the oral route was found to be ineffective *in vivo*, intracranial delivery of 5microlitres of 1millimolar fenofibrate to 5 mice with U-87MG-Luc tumors led to a 6 fold decrease in tumor proliferation compared with DMSO treated controls [69].

Cell survival of LN-229 cells treated with fenofibrate was enhanced where a non-cytotoxic dose of rapamycin was added which could be reflective of enhanced energy release as part of effective autophagy upregulation. Conversely, cell death was enhanced by the addition of chloroquine or bafilomycin which prevent recycled contents from being released out of the autophagsosomes [69].

The use of fenofibrate as part of a combinatorial strategy in pediatric cancer patients has been investigated in a recent phase 2 clinical trial which used a novel approach of continuous low dosing of medications- known as a metronomic strategy- aiming to simultaneously exploit the anti-angiogenic properties of several different pharmaceuticals. The approach demonstrated a partial response (decrease in tumor growth <50%) or stabilization in 8/12 patients with low grade glioma and 12/19 patients with ependymoma, although only 58% and 37% respectively of patients were able to complete the 27 week course of treatment [70].

A further study into the use of fenofibrate in cancer treatment used mouse BsB8 cells, a model for pediatric medulloblastoma. Pediatric medulloblastoma has an incidence of 0.2 per 100,000 in England (1995-2003) [71] and is the most common malignant brain tumor in childhood. High incidence in younger children and quick progression compared to other subtypes of childhood brain tumor [72] make it an essential research topic. 25 micro moles of fenofibrate added to mouse BsB8 cells for 24hrs reduced the phosphorylation of regulators IRS-1, AKT and GSK-3B, which could have the downstream effect of inducing autophagy. There was reduced growth noted in these cells, suggesting that autophagy modulation may also have an effect in medulloblastoma cells [73].

### Enhancement of autophagy induction during radiotherapy induces cell growth arrest in adult CNS cancer

As noted above radiotherapy has been shown to induce autophagy which itself can be cytoprotective. Palumbo *et al.* investigated the role of autophagy in radiotherapy treatment of glioblastoma using radiosensitive T98G cells and in U373MG cells which show limited sensitivity to this treatment. In T98G cells, low levels of radiotherapy resulted in enhanced cell death with increases in the action of autophagy components Beclin 1, ATG5 and enhanced conversion of LC3-I to LC3-II, signifying increased autophagosome maturation. siRNA knockout of essential autophagy genes (Beclin/ATG7) ameliorated the reduction in cell proliferation, suggesting a potential requirement of functional autophagy for cell death. Cell death was enhanced by rapamycin, a compound known to induce autophagy at the early stages. U373MG cells were not affected in their individual viability after treatment with rapamycin but their survival fraction decreased dramatically [66]; an effect possibly linked to the buildup of autophagosomes. Overall, the study highlighted a potential role of functional autophagy in cell death following radiotherapy.

Treatment of radioresistant adult glioblastoma cells with the dual mTOR and P13K inhibitor NVP-BEZ235 (see Figure 1) was found to lead to cell growth arrest and a reduction in tumor proliferation after radiotherapy [74]. NVP-BEZ235 treatment of U251 glioblastoma cell line showed increased autophagy levels as indicated by the enhanced conversion of LC3 I-II. The observed increase in radiosensitisation may have been due to a combination of factors including autophagy activation. It was proposed that NVP-BEZ235 might also interfere with DNA damage repair through the AKT/mTOR pathway. Both rapamycin and mTOR inhibitor PP242 were found to cause irreversible growth arrest on a range of head and neck cancer cells treated with radiotherapy including glioma cells [75], providing further evidence of the potential for pharmacological mTOR manipulation to be a viable treatment mechanism in addition to radiotherapy. It should be noted that enhanced autophagy activation can happen alongside cell death and is not necessarily causative. Further studies are needed to confirm a possible link between autophagosome build up and cell death with possible mechanisms being cell starvation (with nutrients locked inside autophagosomes) or modulation of cell signaling.

## RELEVANCE TO PEDIATRIC BRAIN TUMORS

### Current evidence of autophagy modulation as a treatment strategy in children’s brain tumors

Evidence of autophagy manipulation in childhood brain tumors is lacking; this conclusion is supported from the limited results of a recent literature search as presented in Table 1. As in adults, the effect of autophagy modulation appears to be tumor specific as highlighted in an investigation into sildenafil alongside etoposide treatment where autophagy was studied as a possible mechanism of cell death. Interestingly, inhibition of autophagy initiation via knockout of either Beclin 1 or ATG5 was found to enhance survival of DAOY/D283 cells but was detrimental to the survival of patient derived HOSS1 medulloblastoma cells [31]; thus emphasizing the need for treatments to be tumor specific if autophagy is to be manipulated successfully in childhood tumors as well as those in adults.

**Table 1:** Current evidence of autophagy modulation as a strategy for treating children’s brain tumors

Paper title	Study aims	Model of disease	Modifier	Outcome	Evidence specific to pediatrics
Autophagy inhibition improves chemosensitivity in BRAF(V600E) brain tumors [76].	Evidence in tumor cells+ a case study suggesting cells with BRAF(V600E) mutation are autophagy dependent	WT BT16 and BRAF^V600E^ 794 (ganglioglioma), AM38 and NMC-G1 mutant cells (astrocytoma)	CQ	Reduced tumor viability only in BRAF (V600E) mutation	BRAF(V600E) mutation is important in pediatric central nervous system (CNS) tumors. Case as below.
Autophagy inhibition overcomes multiple mechanisms of resistance to BRAF inhibition in brain tumors [77].	Evidence in cells of LC3 indution with chloroquine in relation to tumor growth	94R and AM38R cells resistant to vemurafenib + multiple case studies.	CQ	Tumor growth reduced. This was also shown with continuing treatment over 2.5 yrs in one case study.	Pediatric case study
PDE5 inhibitors enhance the lethality of standard of care chemotherapy in pediatric CNS tumor cells [31].	Investigation of mechanism of action for cell death after treatment with sildenafil.	DAOY/D283 patient derived HOSS1 medulloblastoma cells treated with etoposide	KO Beclin 1 /ATG5	Enhanced survival of DAOY/D283 cells; reduced survival HOSS1 cells	Pediatric CNS tumor cells
Salinomycin induced ROS results in abortive autophagy and leads to regulated necrosis in glioblastoma [78].	Investigation into mechanism of action of salinomycin against tumor cells	SF188, GSC11 glioblastoma cell lines	Salinomycin	Salinomycin enhances ROS, thus inducing autophagy which was then blocked with build up of lysosomes. Cell death then occurred via necrosis.	Pediatric high grade glioma cells
Restoration of miR-30a expression inhibits growth, tumorigenicity of medulloblastoma cells accompanied by autophagy inhibition. [79].	Effect of miR-30a on autophagy and cell death	DAOY- SHH medulloblastoma D285- type 4-5 D4250 group 3	miR-30a	MirR-30a inhibits autophagy (reduces beclin 1/ATG5 expression) and was linked to increased cell death	DAOY cell line from desmoplastic cerebellar medulloblastoma of a 4 yr old [80].
The p53 tumor suppressor protein protects against chemotherapeutic stress and apoptosis in human medulloblastoma cells [81].	Effect of 3-MA / CQ on survival of D556 and DAOY cells (secondary outcome)	D556, DAOY	3MA /CQ	None	DAOY cell line from desmoplastic cerebellar medulloblastoma of a 4 yr old [80].
Modulation of a brain tumor autophagy and chemosensitivity [9].	Effect of rapamycin/CQ on DAOY + BT-16 CNS atypical teratoid/rhabdoid tumor cells survival +CCNU and cisplatin	DAOY+ ONS76 medulloblastoma cells as well as BT-16+ BT-12 CNS atypical teratoid/rhabdoid tumor cells	Rapamycin/CQ	None	DAOY cell line from desmoplastic cerebellar medulloblastoma of a 4 yr old [80].

Treating children’s brain tumors.

A search in PubMed for the terms “autophagy AND children’s brain tumors/autophagy AND pediatric brain tumors” (11.4.18) returned 35 and 34 results respectively. 13 papers were identified in both of these searches. 11 papers presented evidence of autophagy manipulation on brain tumor models; of which seven presented evidence specific to the pediatric brain tumors rather than adult pathology; with two finding no effect of autophagy modulation on cell survival [9, 81].

Abbreviations: chloroquine, CQ; knockout, KO.

### Evidence of autophagy modulation as a treatment strategy in pediatric brain tumors with V600E mutation

BRAF, a kinase implicated in cell growth and survival, is activated following growth factor-receptor binding [52, 82, 83]. The V600E mutated variant of BRAF disrupts auto-inhibition, leading to constant activation of cell growth and has been identified in various types of pediatric brain tumors. These include on average 9% of pilocytic astrocytomas (one of the most common brain tumors in childhood), 33% of anaplastic astrocytomas and 69% of pleomorphic xanthoastrocytomas. The mutation is generally more abundant in pediatric CNS tumors than those found in adults [82]. CNS tumor cells with the BRAF V600E mutation have higher rates of autophagy in response to cell stress than those without. Studies have found that treatment using growth inhibitor vemurafenib, a chemotherapy agent, combined with chloroquine results in a greater reduction in viability of tumor cells with the mutation than in those without [76]. This phenomenon has also been demonstrated in a case study of a brainstem ganglioglioma with BRAF V600E mutation first presenting at 13 years of age. Treatment of the patient with vemurafenib and vinblastine resulted in manifestations of resistance in contrast to vemurafenib-chloroquine combination which reduced tumor growth over a 2.5 year follow up period [76, 84]. The success of this treatment may have been influenced by the modulation of autophagy through the combined action of chemotherapy and chloroquine in the presence of BRAF V600E mutation.

UAI-201 is another BRAF targeting drug that was found to cause dose-dependent inhibition of glioma growth in cells with V600E mutation, including the KG-1-C line from glioma cells of a 13 year old boy. Treatment of these V600E mutated cells with UAI-201 resulted in an increase in LC3-I to LC3-II conversion indicative of autophagy activation. Deletion of Beclin-1 lessened the anti-proliferative effect of UAI-201 in cells with the V600E mutation [84]. The requirement for Beclin-1 provides good evidence that autophagy is involved in cell death in BRAF mutation (where it is already genetically upregulated). Because BRAF mutation is generally more common in pediatric CNS tumors, than in adult cases the strategy of targeting autophagy holds promise in this group.

### Evidence of autophagy modulation as a treatment strategy for subependymal giant cell astrocytoma in children with tuberous sclerosis

Several genetic mutations can predispose to the development of a low grade glioma. Tuberous sclerosis (TS) is an autosomal dominant condition caused by mutation of either *TSC1* or *TSC2* and is associated with neurological effects such as seizures, autism and reduced intellect as well as tumor formation in the heart, brain, lungs and kidneys [85]. Subependymal giant cell astrocytoma (SEGA) is a benign brain tumor occurring in up to 20% of patients with TS and most commonly occurs between the ages of 10 and 20 years. It can be treated using the antineoplastic chemotherapy drug everolimus; an mTOR inhibitor [86].

Wild type TSC1 and 2 combine to form a complex involved in tumor suppression that inactivates the GTPase Rheb [87], thus decreasing mTOR signaling which relieves mTOR-mediated inhibition of autophagy. Mutation in either *TSC1* or *TSC2* leads to constant activation of mTOR [88] which could suppress autophagy activity. TSC2 negative murine embryonic fibroblasts were found to have smaller and fewer autophagosomes at baseline than wild type cells, with a reduced rate of autophagosome formation. Genetic reduction of *SQSTM1* (coding for autophagy receptor p62) inhibited growth in xenograft tumors with homozygous deletion of TSC2, proving the activity of SQSTM1/p62 and the process of autophagy to be still somewhat active in these tumors. However, autophagy inhibition remains a challenge in this case due to lack of selectivity for tumor cells. SEGA tumor growth was inhibited through using a combination of rapamycin and chloroquine where rapamycin induces the formation of autophagosomes whilst chloroquine inhibits their destruction [88]. In support of this model the drugs were found to be more cytotoxic in combination than when used alone. These results are noteworthy as they demonstrate that autophagy modulation may be of use even in the absence of additional genetic/pharmacological autophagy induction.

### CONCLUDING REMARKS

Evidence of autophagy manipulation in pediatric brain tumors is limited and is tumor-specific. However, manipulation of autophagy remains an exciting candidate treatment strategy, especially alongside chemotherapy, where cancerous cells with high turnover are likely to have been affected by cytotoxic agents and may be undergoing autophagic removal of damaged proteins and organelles. The resulting up-regulation of autophagy in tumor cells following treatment could potentially make them more vulnerable to modulation than in healthy tissue. Disease-associated genetic mutation can have an effect on baseline cellular autophagy rates as demonstrated in V600E mutation of BRAF and this could make autophagy modulation particularly useful in affected cells. However, cytotoxicity through combinatorial use of several agents to manipulate autophagy can be achieved even in tumors with genetic downregulation of autophagy, as in the case of *TSC* mutations.

## FUTURE PERSPECTIVE

Childhood brain tumors continue to be the leading cause of cancer-related death in this age group and are, therefore, an important area of research currently and in the future. Developing medication is particularly important for childhood brain tumors as strategies to both improve survival and to minimize therapy related long term sequelae are so clearly needed. Although autophagy action is influenced by both genetic and environmental factors, use of the combination approach is not necessarily limited in the absence of constitutive autophagy up-regulation and could, therefore, potentially be investigated as an adjunct to current therapy in all types of childhood brain tumor.

## EXECUTIVE SUMMARY

•Childhood brain tumors currently represent a significant research area.
•Autophagy is a process already implicated in tumor evolution, and leads to stage-dependent enhancement or reduction of tumor growth.
•Autophagy can be upregulated in tumor cells undergoing chemo and radiotherapy.
•The regulation of autophagy can already be targeted at specific stages using available drugs.
•The autophagy inhibitor chloroquine has been shown to reduce glioblastoma growth.
•Induction of autophagy induces cell growth arrest in adult CNS tumors being treated with radiotherapy.
•There is some evidence of autophagy modulation as a successful treatment strategy in pediatric brain tumors. This so far includes those with the V600E mutation and SEGA tumors in tuberous sclerosis.
